# Evidence for genetic variance in resistance to tuberculosis in Great Britain and Irish Holstein-Friesian populations

**DOI:** 10.1186/1753-6561-5-S4-S15

**Published:** 2011-06-03

**Authors:** Mairead L Bermingham, Susan Brotherstone, Donagh P Berry, Simon J More, Margaret Good, Andrew R Cromie, Ian MS White, Isabella M Higgins, Mike Coffey, Sara H Downs, Elizabeth J Glass, Stephen C Bishop, Andy P Mitchell, Richard S Clifton-Hadley, John A Woolliams

**Affiliations:** 1 The Roslin Institute, Roslin Biocentre, Roslin, Midlothian EH25 9RG, UK; 2 Institute of Evolutionary Biology, University of Edinburgh, West Mains Road, Edinburgh EH9 3JT, UK; 3 Moorepark Production Research Centre, Fermoy, Co. Cork, Ireland; 4 Centre for Veterinary Epidemiology and Risk Analysis, UCD School of Agriculture, Food Science and Veterinary Medicine, University College Dublin, Belfield, Dublin 4, Ireland; 5 Department of Agriculture, Fisheries and Food, Kildare St., Dublin 2, Ireland; 6 The Irish Cattle Breeding Federation, Bandon, Co. Cork, Ireland; 7 Scottish Agricultural College, Bush Estate, Penicuik, Midlothian EH26 0PH, UK; 8 Veterinary Laboratories Agency-Weybridge, New Haw, Addlestone, Surrey KT15 3NB, UK

## Abstract

**Background:**

Here, we jointly summarise scientific evidence for genetic variation in resistance to infection with *Mycobacterium bovis*, the primary agent of bovine tuberculosis (**TB**), provided by two recent and separate studies of Holstein-Friesian dairy cow populations in Great Britain (**GB**) and Ireland.

**Methods:**

The studies quantified genetic variation within archived data from field and abattoir surveillance control programmes within each country. These data included results from the single intradermal comparative tuberculin test (**SICTT**), abattoir inspection for TB lesions and laboratory confirmation of disease status. Threshold animal models were used to estimate variance components for responsiveness to the SICTT and abattoir confirmed *M. bovis* infection. The link functions between the observed 0/1 scale and the liability scale were the complementary log-log in the GB, and logit link function in the Irish population.

**Results and discussion:**

The estimated heritability of susceptibility to TB, as judged by responsiveness to the SICTT, was 0.16 (0.012) and 0.14 (0.025) in the GB and Irish populations, respectively. For abattoir or laboratory confirmation of infection, estimates were 0.18 (0.044) and 0.18 (0.041) from the GB and the Irish populations, respectively.

**Conclusions:**

Estimates were all significantly different from zero and indicate that exploitable variation exists among GB and Irish Holstein Friesian dairy cows for resistance to TB. Epidemiological analysis suggests that factors such as variation in exposure or imperfect sensitivity and specificity would have resulted in underestimation of the true values.

## Background

Tuberculosis (**TB**) is a serious disease of cattle caused by *Mycobacterium bovis*. The disease can be transmitted to humans by close contact with infected livestock or their tissues or secretions, or through ingestion of either contaminated milk or milk products. In order to reduce zoonotic risk, a TB control test and slaughter policy was introduced in Great Britain (**GB**) and Ireland in the 1950s. During the initial stages of the program, progress was rapid, leading to a considerable reduction in the incidence of the disease in cattle. However, since the mid-1960s progress in control has stalled, and the herd incidence of TB in 2006 was 6.87% and 5.72% in GB and Ireland, respectively [1]. The persistence of TB in GB and Irish cattle populations has been attributed to a range of factors, particularly ongoing wildlife-to-cattle and cattle-to-cattle transmission [2]. The failure of GB and Ireland to reach TB-free bovine herd status, coupled with the relatively high cost of the existing control programs, indicates a need to investigate alternative strategies. One approach, which could complement current control programmes, would be genetic selection for increased resistance to TB in cattle, exploiting potential between-animal variation in TB resistance. This paper summarises the scientific evidence for genetic variation in resistance to infection with *M. bovis* provided by two recent and separate studies of Holstein-Friesian dairy cow populations in the GB and Ireland [3,4]. The aim is to assess the reproducibility of the genetic parameters estimated from two genetically distinct populations using different methodological and analytical approaches.

## Methods

### Ante mortem surveillance of TB

The tuberculin test is the international standard for ante mortem diagnosis of bovine TB in cattle [5]. *Mycobacterium avium* and other *Mycobacterium* spp. are prevalent in the environment in GB and Ireland, causing non-specific sensitization to *M. bovis* tuberculin. Thus, the single intradermal comparative tuberculin test (**SICTT**) is used, involving separate intradermal injections of *M. bovis*-purified protein derivative (**PPD**) and *M. avium*-PPD antigens. This test works on the premise that *M. bovis*-infected cattle tend to show a greater response to *M. bovis*-PPD than to *M. avium*-PPD [5]. The SICTT measures the difference in skin thickness in response to the *M. bovis*-PPD and M. *avium*-PPD 72 h after injection [6]. In Ireland, all herds are routinely tested at 12-month intervals, whereas in GB testing takes place every 12-48 months according to the regional herd incidence of TB over the previous 2, 4 or 6 years [5]. Under the ‘standard’ interpretation of the SICTT, a standard reactor (**R**) is an animal with a skin change in response to the *M. bovis*-PPD inoculation 4mm or greater than the reaction to the *M. avium*-PPD inoculation. Whereas, an inconclusive reactor (**IR**) is an animal with a skin change in response to the M. *bovis*-PPD inoculation larger than 0 mm but less than 4 mm greater than the reaction to the M. *avium*-PPD inoculation [7]. All R are culled in GB and Ireland. Whereas, IR undergo further testing; until the third IR result in GB or the animal is removed as a reactor under, ‘severe’ interpretation of the SICTT in Ireland. Discovery of a R triggers a herd breakdown during which the herd is placed under movement restrictions until it is officially considered free of TB.

### Post-mortem surveillance of TB

Post-mortem examination of every animal at slaughter, as part of the ongoing bovine TB control programmes in GB and Ireland, enables diagnosis of disease status. Furthermore, lesions from *M. bovis* non-reactor animals at slaughter are sent for histological analysis. Together, these data enabled confirmation of TB breakdowns and provided animal-level data in both studies.

### Compilation of datasets

Animal level SICTT results and abattoir animal lesion records from 1995-2007 and 2000-2005 were extracted from the GB Defra VetNet database and the Irish Department of Agriculture, Fisheries and Food (**DAFF**) Animal Health Computer System (**AHCS**), respectively. Non reactor animals at a SICTT are recorded in the Irish (but not the GB) database. Dairy cow records and non-reactor SICTT contemporaries were extracted using the GB monthly milk yield records collected closest in time to the SICTT. Dairy cow records in the Irish study were extracted using birth, calving and calving interval records. Both datasets were edited to maximize the opportunity of exposure to *M. bovis* between and within breakdowns. Only breakdowns with at least two Rs were included in the analysis because the probability of two false positives is minimal [2]. The requirement for one home-bred R in the Irish study ensured that exposure was not transient exposure from the entry of an *M. bovis* infected animal. Furthermore, cows that moved into the herd within six weeks of the herd test in the Irish study were removed because insufficient time had lapsed to develop a positive reaction to the SICTT post-exposure [5]. In the GB study, based on the assumption that the breakdown originated from a point source within a cohort, all other age groups other than the standard reactor cohort were deleted. Following all edits, 68,497 SICTT records and confirmed *M. bovis* infection records were available from 818 breakdowns for inclusion in the GB study; and 14,013 SICTT records from 436 breakdowns and 13,791 confirmed *M. bovis* infection records from 392 breakdowns were available in the Irish study.

### Statistical analysis

SICTT responsiveness in the GB study was classified depending on whether a cow had been culled (irrespective of whether it was culled as a R or three-fold IR) during, or survived to the end of the breakdown. Whereas, in the Irish study a high diagnostic threshold for a standard reactor was set, and IR were removed from the analysis of SICTT to reduce the likelihood of false positives or negatives. SICTT responsiveness in the Irish study was dichotomized as R and non-reactor.

Confirmed *M. bovis* infection in the GB study was classified depending on whether a cow had been culled and had gross pathological or bacteriological evidence of infection, or survived to the end of breakdown. In the Irish study, confirmed *M. bovis* infection was simply dichotomized as lesioned and non-lesioned cows. All major effects likely to be associated with the SICTT [2], including month and year of SICTT, lactation group, age within lactation group (Irish study) and milk yield (GB study), were included in the statistical models.

Threshold models were used to analyse susceptibility to *M. bovis* infection. The coefficients from a binary response model with complementary log-log link function have the same relative risk interpretation as from survival analysis using Cox proportional hazards model [8]. A complementary log-log model was therefore chosen to analyse the survival data from the GB study. Threshold models which assume errors following a logistic distribution converge more easily; threshold models assuming a logistic underlying liability variable, fitted using a logit link function were therefore used to analyse the trait measures from the Irish study.

## Results and discussion

**Figure 1:**
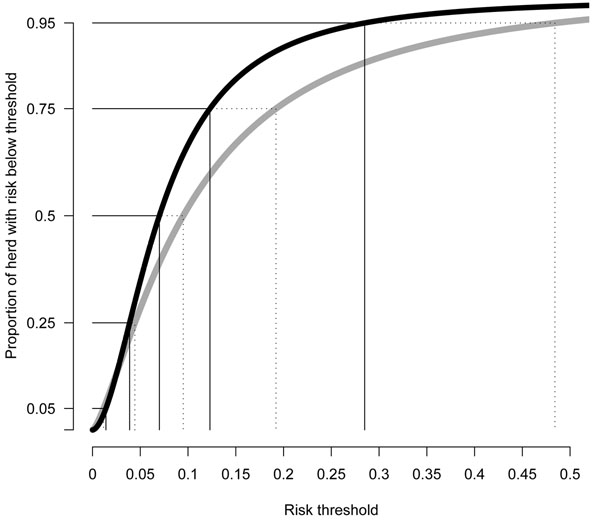
**Cumulative distribution of individual risk in herd breakdowns derived from a normal approximation to the models fitted, within the GB (black) and Irish (grey) datasets**. x-axis = risk threshold, y-axis = proportion of animals in the herd with risk below the threshold.

Individuals within herd breakdowns were found to have differing probabilities of responding to the SICTT in the two studies (Figure 1). In the GB study the most susceptible five percent of the cows had an estimated risk of ≥0.29 of being culled as a reactor, whereas in the Irish study the equivalent group of cows had a somewhat higher estimated risk, ≥0.48, of exhibiting a standard reaction to the SICTT. This may be an indication that the cows in the GB study were less likely to respond to the SICTT following exposure to *M. bovis*, or that the Irish cows had a greater opportunity for exposure (average herd breakdown reactor prevalence was 7% in the GB data set, as compared to 10% standard reactor prevalence in the Irish dataset) and hence had a greater opportunity for trait expression than the GB cows.

Significant genetic variation for SICTT responsiveness and susceptibility to confirmed *M. bovis* infection exists within the GB and Irish Holstein-Friesian dairy herds. Irrespective of the differences in TB prevalence, level of exposure, risk factors accounted for, trait definition, or models employed, estimates of the heritability (additive genetic variance as a proportion of total variance) for confirmed *M. bovis* infection were almost identical at 0.18 (0.044) and 0.18 (0.041), and estimates of the heritability for responsiveness to the SICTT were similar at 0.16 (0.012) and 0.14 (0.025) from the GB and the Irish populations, respectively.

The heritability estimates for responsiveness to the SICTT and susceptibility to confirmed *M. bovis* infection were similar to, or slightly higher than, those in the literature for TB resistance [0.06 to 0.08; 9] and other related disease traits such as paratuberculosis [0.06, 10; 0.07-0.15, 11]. Furthermore, higher heritabilities were estimated for the TB trait measures in GB and Irish studies as compared with clinical mastitis (0.094; 12); even with low heritabilities clinical mastitis is directly or indirectly (via reduced somatic cell count) incorporated into breeding programmes in many countries.

Theoretically, the true heritability for responsiveness to the SICTT in Holstein-Friesians may be much higher, as inaccuracies resulting from imperfect SICTT sensitivity and specificity across herd breakdowns [5] may have resulted in underestimation of the heritability estimates. The estimates of sensitivity and specificity from the literature are similar in the current GB and Irish TB control programmes [5]. Results from a recent exploration of the genetic properties of disease data [13] suggest that, despite the similarity of the diagnostic characteristics of the SICTT under GB and Irish conditions, the greater herd incidence of TB in GB combined with likely incomplete exposure of animals to infection may have resulted in the GB heritability estimates having a greater downward bias than the Irish estimates. Using the imperfect diagnostic test adjustment formula for observed heritability [13]; the predicted true heritabilities for *M. bovis*-PPD responsiveness in the GB and Irish studies were 0.22 and 0.18 respectively. The development of genetic epidemiological models that better describe levels of exposure/infection pressure within breakdowns will provide greater power to meaningfully quantify genetic variation, and hence allow greater comparative inferences to be drawn regarding the level of exploitable genetic resistance to TB that exists in the GB and Irish Holstein-Friesian populations.

## Conclusions

The GB and Irish studies have undoubtedly demonstrated that significant genetic variation for susceptibility to confirmed *M. bovis* infection exists among GB and Irish Holstein-Friesian dairy cows. The large, high-quality VetNet and AHCS databases, the methodological and analytical equalization of risk of failing the SICTT and exposure to *M. bovis*, the application of statistically appropriate threshold models, and corroborative estimates from the two studies have provided credible and robust heritability estimates for responsiveness to the SICTT and susceptibility to confirmed *M. bovis* infection in cattle. These data are collected routinely within the national TB control programs in GB and Ireland. Therefore, it should be possible to develop breeding programs to select for resistance to TB in GB and Irish Holstein-Friesian populations.

## List of abbreviations used

TB tuberculosis ; GB Great Britain; SICTT single intradermal comparative tuberculin test ; PPD purified protein derivative ; R standard reactor ; IR inconclusive reactor ; DAFF Department of Agriculture, Fisheries and Food ; AHCS Animal Health Computer System

## Competing interests

The authors declare that they have no competing interests.
